# Large-scale insertional mutagenesis of a coleopteran stored grain pest, the red flour beetle *Tribolium castaneum*, identifies embryonic lethal mutations and enhancer traps

**DOI:** 10.1186/1741-7007-7-73

**Published:** 2009-11-05

**Authors:** Jochen Trauner, Johannes Schinko, Marcé D Lorenzen, Teresa D Shippy, Ernst A Wimmer, Richard W Beeman, Martin Klingler, Gregor Bucher, Susan J Brown

**Affiliations:** 1 Department of Biology, Developmental Biology, Friedrich-Alexander-University Erlangen, Erlangen, Germany; 2 Department of Developmental Biology, Johann-Friedrich-Blumenbach-Institute of Zoology and Anthropology, Georg-August-University Göttingen, GZMB, Ernst-Caspari-Haus, Göttingen, Germany; 3 USDA-ARS-GMPRC, Manhattan, KS, USA; 4 Division of Biology, Ackert Hall, Kansas State University, Manhattan, KS, USA

## Abstract

**Background:**

Given its sequenced genome and efficient systemic RNA interference response, the red flour beetle *Tribolium castaneum *is a model organism well suited for reverse genetics. Even so, there is a pressing need for forward genetic analysis to escape the bias inherent in candidate gene approaches.

**Results:**

To produce easy-to-maintain insertional mutations and to obtain fluorescent marker lines to aid phenotypic analysis, we undertook a large-scale transposon mutagenesis screen. In this screen, we produced more than 6,500 new *piggyBac *insertions. Of these, 421 proved to be recessive lethal, 75 were semi-lethal, and eight indicated recessive sterility, while 505 showed new enhancer-trap patterns. Insertion junctions were determined for 403 lines and often appeared to be located within transcription units. Insertion sites appeared to be randomly distributed throughout the genome, with the exception of a preference for reinsertion near the donor site.

**Conclusion:**

A large collection of enhancer-trap and embryonic lethal beetle lines has been made available to the research community and will foster investigations into diverse fields of insect biology, pest control, and evolution. Because the genetic elements used in this screen are species-nonspecific, and because the crossing scheme does not depend on balancer chromosomes, the methods presented herein should be broadly applicable for many insect species.

## Background

During the past few years, the red flour beetle *Tribolium castaneum *has been developed into a powerful model organism suited for the study of short germ development, embryonic head and leg development, metamorphosis, cuticle metabolism, and other problems in insect biology. It is the first coleopteran species for which the genome sequence has become available [1]. In-depth functional analysis of molecularly identified genes is enabled by the availability of germline transformation [2,3] and systemic RNA interference that is splice-variant-specific [4] and feasible at all life stages [5-7]. Furthermore, several tools and techniques have been developed that facilitate insertional mutagenesis in *Tribolium castaneum *[8-11]. Although candidate gene approaches (reverse genetics) via RNA interference work very well in *Tribolium*, they are biased towards previously recognized genes and mechanisms. In contrast, forward genetic approaches offer the opportunity to detect new gene functions not yet described in other model systems. Small-scale chemical mutagenesis screens have been performed in *Tribolium *[12,13], but stock-keeping of unmarked recessive mutants is difficult due to the number of chromosomes (n = 10) and the lack of balancers (< 50% of the genome is covered) [14]. In contrast, insertional mutagenesis screens using dominantly-marked *donor *transposons facilitate both stock-keeping and gene identification.

Several species-nonspecific transposons including *Hermes*, *Minos*, and *piggyBac *have been shown to function in *Tribolium *[2,10]. Berghammer et al. [2] introduced enhanced green fluorescent protein (EGFP) under the control of the 3xP3 promoter as a universal, selectable marker for transgenic insects. This promoter is also responsive to nearby chromosomal enhancers [3], allowing insertional mutagenesis to be combined with enhancer trapping [9]. In our scheme, insertional mutagenesis is based on the controlled remobilization of a non-autonomous donor element stably inserted in the genome. The transposase needed to remobilize the donor element is provided by a helper element (*jumpstarter*). Lorenzen et al. [11] created several jumpstarter strains using a modified *Minos *transposon to provide a source of *piggyBac *transposase [9].

Here we report the first large-scale insertional mutagenesis screen conducted in an insect other than *Drosophila*. We have identified many insertion lines that are either homozygous lethal, homozygous sterile and/or show enhancer-trap patterns at various developmental stages. The genomic locations, enhancer-trap patterns (if present), recessive phenotypes, and genes affected by these transposon insertions are documented in the GEKU database (freely accessible at http://www.geku-base.uni-goettingen.de) and insertion lines are available upon request [GEKU: Göttingen, Erlangen, Kansas State University (KSU), United States Department of Agriculture (USDA)].

Our screening procedure should be applicable to many other insect species, because all genetic elements (transposons, promoters and marker genes) used in this screen are species-nonspecific [9]. It also renders unnecessary the use of balancer chromosomes, which are not available for the vast majority of insect species. Obvious limitations may be the ability to rear the insect species in the laboratory, the feasibility of germline transformation to obtain donor and helper strains, and the ability to perform single-pair matings with high efficiency.

## Results

### Test for lethality and sterility

**Figure 1:**
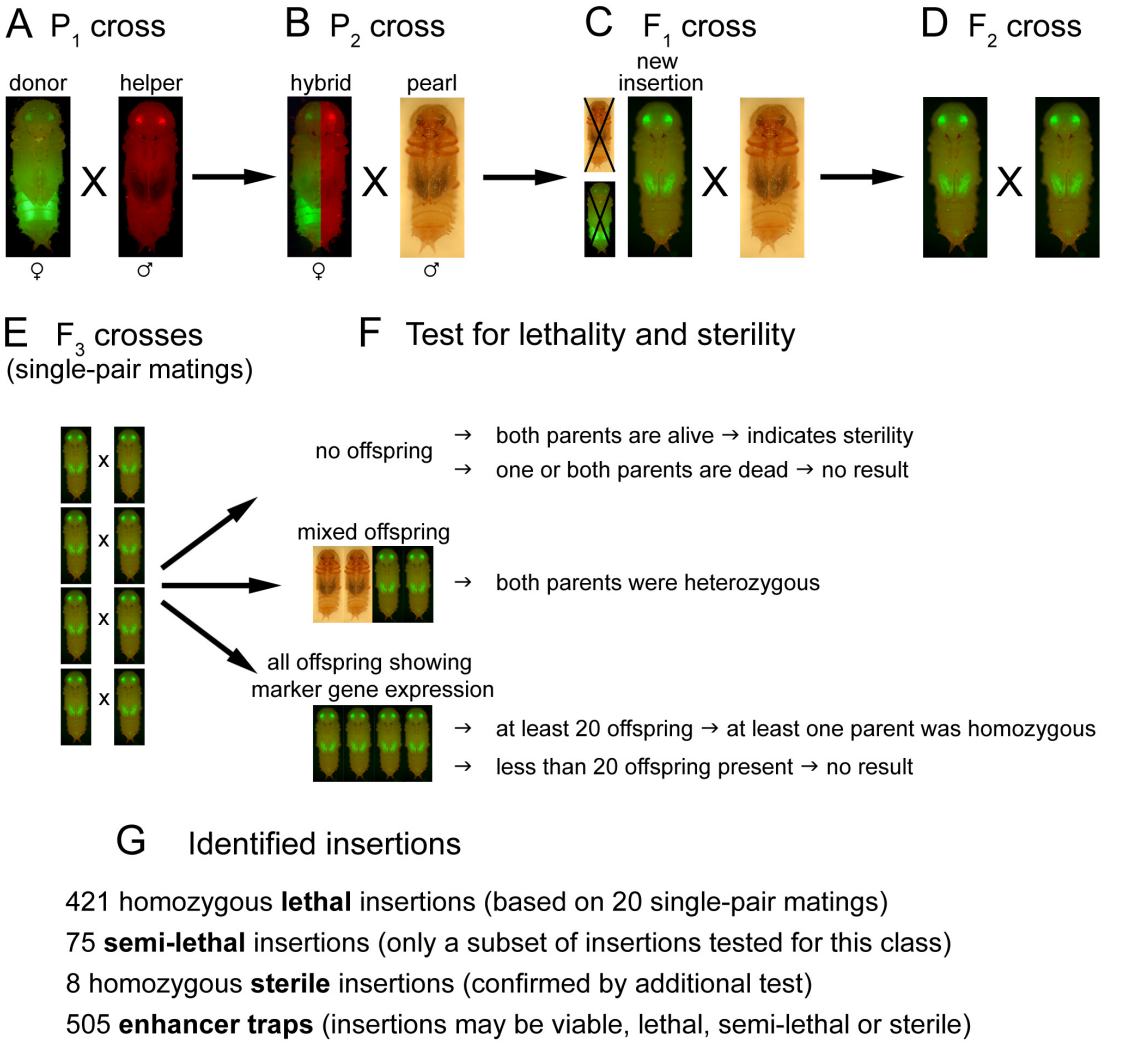
**Screening procedure for the creation of new insertions and identification of lethal and sterile mutations**. **(A) **P_1 _cross: Mass-crosses were made between the donor (EGFP-marked eyes) and the helper strain (DsRed-marked eyes). The donor strain has an additional insertion site-dependent, muscle-specific enhancer-trap pattern. **(B) **P_2 _cross: Single hybrid females carrying both the donor and helper elements (simultaneous expression of EGFP and DsRed) were crossed to three *pearl *males. **(C) **F_1 _cross: A single individual carrying a stable new insertion was selected from the offspring of a P_2 _cross and crossed to several *pearl *mates. A remobilization event was evident in beetles that still showed EGFP-marked eyes, but had lost the muscle-specific enhancer-trap pattern. Note the altered enhancer-trap phenotype of the new insertion line in this example (EGFP expression in the wings). **(D) **F_2 _cross: All EGFP-marked offspring of the F_1 _cross were heterozygous for the insertion and were sibling-crossed. **(E) **F_3 _cross: Several single-pair matings were set up. **(F) **Test for lethality and sterility: Marker gene expression of the offspring of each single-pair mating was evaluated to determine whether their parents had been hetero- or homozygous for the *piggyBac *insertion (see Methods). Each single-pair mating was assigned to one of five classes (small black arrows; Table 4). The combined evaluation of all single-pair matings was used to define the phenotype of the insertion (see Table 5). **(G) **Summary of all identified insertions. For further details see methods part "Generating new *piggyBac *insertions", Table 1, and text.

Following the procedure illustrated in Figure 1, a total of 6,816 new, independently derived insertions were isolated in the F_1 _generation and of these, 5,657 new insertion lines were successfully tested for lethality/sterility. 589 potentially homozygous lethal lines were identified in the first round of F_3 _crosses, of which 421 (i.e. 7.4% of 5,657 insertions) were confirmed to be homozygous lethal in the second round (Table 1; for details on the two rounds of screening F_3 _crosses please see Methods). A subset of the viable insertion lines, those producing fewer homozygotes than expected, was tested for semi-lethality. Insertion lines were designated as potentially semi-lethal if homozygosity of a parent was indicated for no more than one single-pair mating in the first round of F_3 _crosses, or less than four single-pair matings after the second round. This was true for 236 insertions (out of the subset of 2,940 insertions analyzed in Göttingen and Erlangen) after the first round, of which 75 remained in this category after the second round. Hence, 2.5% (75/2,940) of all insertions tested for semi-lethality met the criteria for semi-lethality. This somewhat relaxed scoring criterion reduced the likelihood of missing or overlooking lethal or semi-lethal mutations.

**Table 1:** Results of lethality/sterility test (F_3 _cross)

First round of single-pair matings		Second round of single-pair matings	
**phenotype**	**number of insertions**	**phenotype**	**number of insertions**
viable*	4908 (86.8%)	viable	250 (4.4%)
potentially lethal	589 (10.4%)	lethal	421 (7.4%)
potentially sterile	160 (2.8%)	sterile	8 (0.1%)
		*not retested*	70 (1.2%)

A total of 5,657 lines were tested for potential lethality or sterility by a first round of single-pair matings (left half of table; see Results and Methods for details). Those that matched the criteria (749) were retested by a second round of additional single-pair matings in order to eliminate false positives (right half of table). Only those lines that matched the definition in the second round were considered to be lethal or sterile. All percentages are calculated based on the original total of 5,657 lines.

*This number includes 236 lines that were considered potentially semi-lethal (see text for definition of semi-lethality). Because this was done only on a subset of 2,940 lines, the numbers are not given separately.

Potentially homozygous sterile insertions lines were identified by evaluating the single-pair matings: Whenever two or more of the initial single-pair F_3 _self-crosses (round one, Figure 1E) failed to produce offspring (although the parents were alive and healthy), the line was classified as potentially sterile. This was the case for 160 insertions (Table 1). We used either of two methods to confirm or refute a tentative diagnosis of recessive sterility. In the first method, we set up a second round of single-pair self-crosses bringing the total number of F_3 _crosses to 20. The diagnosis was considered to be corroborated when the number of single-pair matings not producing any offspring increased to four or more. Using this definition, 124 potentially sterile lines were reduced to 21. However, further testing of these presumably sterile insertion lines showed that this criterion was not always reliable (see below). In the second method we set up 10 male and 10 female outcrosses. The diagnosis of recessive sterility was considered to be corroborated if the crosses failed to reveal either a fertile, homozygous male or a fertile, homozygous female. Out of 36 potentially sterile lines tested by the second method, only eight lines fulfilled this definition of sterility. Since the second follow-up test appeared to be more rigorous than the first, we retested 11 of the 21 apparently sterile lines from the former test using the more rigorous criterion. All 11 lines proved to be fertile in both sexes. It seems to be clear that most sterile lines found by using the first criterion are false-positives. Hence, we suggest using the stricter test for recessive sterility, which has the added benefit of identifying the affected sex.

### Detection of enhancer traps

**Figure 2:**
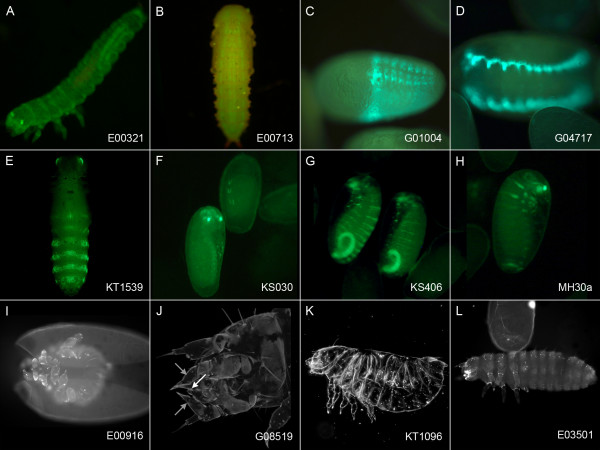
**Examples for enhancer traps and mutant phenotypes**. Enhancer traps (A-H) and mutant phenotypes (I-L) of *piggyBac *insertion lines. For details on the potentially affected genes, see methods part "Location of insertion lines shown in Figure 2". (A) EGFP expression in the cuticle during all larval stages. (B) Pupa showing EGFP expression in a subset of somatic muscles. (C) Embryo showing EGFP expression in the abdomen. (D) Embryo showing EGFP expression in two lateral stripes, which based on the similarity to the *Drosophila *expression pattern of *lame duck *is presumably located in the mesoderm. (E) Pupa showing EGFP in a "salt and pepper" pattern in the ventral abdominal epidermis. (F) Embryos showing EGFP expression in the distal legs. (G) Embryos showing EGFP expression in the hindgut and in segmental stripes. (H) EGFP expression is in the proximal embryonic leg, as well as in T2, T3, and A1 spots, and a posterior ring in the first-instar larva. (I) Homozygous embryo is poorly differentiated and has bubbly short legs and segmental defects. (J) Maxillary (grey arrows) and labial (white arrow) palps are transformed to legs while the overall morphology of the segments appears unchanged (this corresponds to the described *Tc-maxillopedia *mutant phenotype [16,17]). (K) Homozygous embryonic cuticle showing dorsal defects and possibly additional patterning or differentiation problems. (L) Homozygous embryo with rudimentary appendages in the first abdominal segment which also lacks tracheal openings.

3xP3-driven EGFP expression is typically seen only in the eyes and central nervous system [3]. We analyzed all new insertion lines for additional, i.e. enhancer-dependent EGFP expression, and detected novel patterns at all developmental stages. Although we observed a bias for certain patterns (i.e. certain central nervous system patterns, segmentally-repeated stripes in embryos, or small dots at the hinges of extremities in larvae and pupae), we identified 505 unique enhancer-trap patterns. The bias for certain patterns might be caused by a favored expression in certain tissues due to the paired-class homeodomain binding sites in the 3xP3 element of the transformation marker [15]. For a random subset of about 200 of all newly-identified insertions, we also dissected pupae and adults to look for EGFP expression in internal organs that might not be visible without dissection. Such expression patterns (e.g. a spermatheca enhancer) were found only rarely. Examples of enhancer-trap lines are shown in Figure 2A-H. Descriptions and/or photographs of all enhancer-trap lines together with information about their chromosomal locations (when known) are available in GEKU-base (http://www.geku-base.uni-goettingen.de; see Methods).

### Analysis of lethal lines and developmental phenotypes

We analyzed the embryonic cuticle phenotypes of many lines identified as lethal and found a number of distinct cuticular abnormalities (Figure 2I-L). For example, line G08519 displays a phenotype similar to the *proboscipedia *ortholog *maxillopedia *in that maxillary (grey arrows) and labial (white arrow) palps are transformed to legs (Figure 2J); [16,17]. Indeed, this insertion is located in the first intron of *maxillopedia*. In addition, many lethal lines showed a high proportion of embryos that died prior to cuticularization, indicating early embryonic lethality.

To test whether the *semi-lethal *lines are false positives or true lethals with occasional *escapers*, we checked what portion of these lines (Göttingen subset) produce lethal L1 cuticle phenotypes (at least two cuticles with similar strong defects in one preparation when scoring at least 10 individuals) and compared it to the percentage of cuticle phenotypes produced by the other classes. A total of 25.8% (8/31) of a random selection of lines complying with the strict definition of lethality showed such phenotypes. Of lines with one or two single-pair matings (out of 20) indicating homozygosity (semi-lethality), this portion was 16.6% in each case (5/30 and 3/18, respectively). Lines with three single-pair matings indicating homozygous viability gave rise to cuticle phenotypes in only 6.25% (1/16). Thus analyzing *semi-lethal *lines led to the identification of additional cuticle phenotype-inducing mutations.

### Chromosomal location of new *piggyBac *insertions

**Figure 3:**
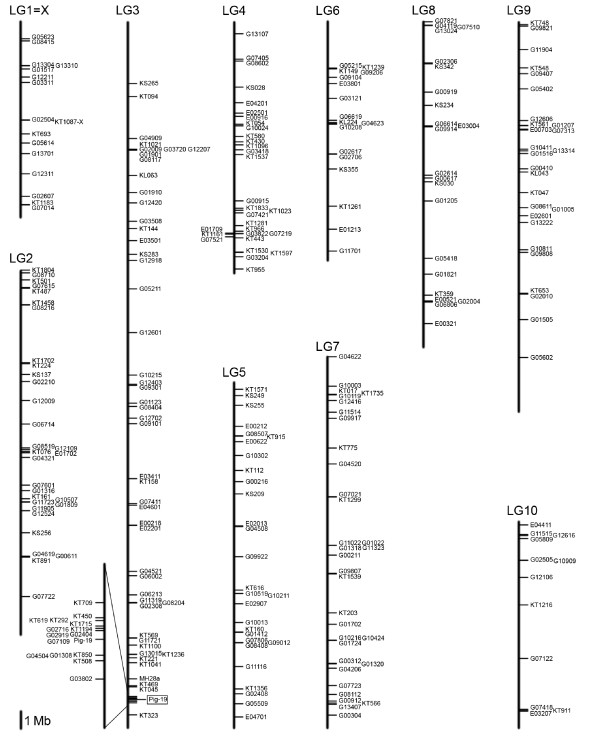
**Distribution of lethal insertions**. Distribution of 280 lethal *piggyBac *insertions on linkage groups 1 to 10. Location of the donor line Pig-19 on LG3 is indicated. Detail of LG3 is magnified 12×. Scale bar = 1 Mb.

We determined the chromosomal location of 400 *piggyBac *insertions by BLAST analysis of amplified flanking sequences against the *Tribolium *genome (see Methods). These insertions included lethal, semi-lethal and sterile as well as viable lines that showed an enhancer-trap pattern. The distribution of 280 homozygous lethal insertions on the linkage groups is shown in Figure 3. The lethal insertions appear to be distributed randomly among the linkage groups, showing a range from 1.1 insertions per Mb for linkage group 10 up to 2.2 insertions per Mb for linkage group 4 (Table 2). Superimposed on the generally random pattern of insertion site locations, there appear to be insertion hotspots and coldspots, the most evident example being the hotspot for local reinsertion near the donor site on linkage group 3.

**Table 2:** Chromosomal location of lethal *piggyBac *insertions

Chromosome	Insertions	Chromosome size* [Mb]	Insertions/Mb
X	**19**	10.9	1.7
2	**31**	20.2	1.5
3	**67**	39.0	1.7
4	**30**	13.9	2.2
5	**31**	19.1	1.6
6	**18**	13.2	1.4
7	**33**	20.5	1.6
8	**22**	18.0	1.2
9	**27**	21.5	1.3
10	**13**	11.4	1.1
unmapped	**14**		

305 insertions were localized in the genome sequence. Of these, 14 were on unmapped scaffolds and 11 could be assigned to chromosomes, but not to the exact position. The distribution of the remaining 280 lethal insertions in the genome is shown in Figure 3.

*based on NCBI map viewer, build 2.1.

## Discussion

The GEKU insertional mutagenesis screen was designed to meet the following criteria: It should be rapid and simple (i.e. involve as few generations as possible); and the analysis of the resulting insertion lines should be highly efficient (i.e. producing only a small number of false positive lethal or sterile lines, while also minimizing the frequency of false negatives; see Methods).

### Large-scale insertional mutagenesis is feasible in a coleopteran species

Based on a pilot screen published in Lorenzen et al. [11] we have performed the first high-efficiency, large-scale insertional mutagenesis screen in an insect species outside the genus *Drosophila*, and we have established a crossing scheme that circumvents the need for balancer chromosomes or embryo injections. From our experience, we estimate that using the procedure presented here, one person should be able to establish 150 lethal strains per year. While the GEKU screen has identified many interesting enhancer traps and lethal phenotypes, genome-wide saturation would be difficult to achieve at the current level of efficiency. The most time-consuming step is setting up and evaluating 20 single-pair matings for each new insertion line to detect recessive lethality. For this reason we set up a small number of single-pair matings first, as most viable insertions can be identified by evaluating just a few crosses from each subset. However, also for insertions recognized as viable it was important to assess the fertility of all remaining single-pair matings in order to ensure that recessive sterile insertions were detected.

### Lethal insertions are readily detected while insertions causing sterility are difficult to detect

We found that lethal lines were readily detected by single-pair matings. Based on the frequency with which semi-lethal lines produced strong L1 larval cuticle phenotypes, we suggest defining lines as potentially lethal when only one or two out of 20 single-pair matings indicate homozygosity. However, our definition of sterility proved to be too lax in the beginning, since most potentially sterile lines turned out to be false-positives in more detailed analysis.

### Comparing efficiencies with *Drosophila melanogaster *insertional mutagenesis and enhancer-trap screens

The efficiency of generating lethal mutations by *piggyBac*-based insertional mutagenesis in *Tribolium *(7.4%) is similar to equivalent screens in *Drosophila *based either on *piggyBac *[9,18] or *P elements *[19-21]. Whether the efficacy of such screens can potentially be doubled by the inclusion of splice acceptor sites or insulator sequences within the mutator element - as has been shown in *Drosophila *[22] - still has to be determined in *Tribolium*.

The enhancer detection rate within this large-scale insertional mutagenesis screen was also 7.4%. This is actually higher than in a comparable *Drosophila *screen where enhancer detection without a suitable amplification system was about 2% [9]. Only after including a GAL4-based amplifier system could *Drosophila *enhancer detection be raised to 50% [9]. However, such directed expression systems still need to be further developed and assayed in *Tribolium *before they can be used in insertional mutagenesis screens.

### Correlation of phenotype (lethality, sterility, enhancer trap) with insertion site proximity to protein coding sequences (CDS)

In 14% of all lethal insertions, *piggyBac *had clearly jumped into the coding sequence of a gene. However, the majority of lethal insertions (61%; see Table 3) were located in introns, apparently disrupting transcription or splicing of the affected gene. One possibility is that the SV40 UTR in the transposon, which serves as a terminator of transcription in both directions, causes early transcriptional termination of the host gene. The tendency of *piggyBac *to insert into intronic sequences had already been observed in *Drosophila *insertional mutagenesis screens [18,22].

**Table 3:** Detailed analysis of lethal *piggyBac *insertion sites

location	number	%
Intron	185	61
CDS*	42	14
< 500 bp**	27	9
500 bp - 2500 bp**	24	8
distant (> 2500 bp)**	27	9
all localized	305	100
seq or blast problem	54	
not sequenced	62	
all lethal	421	

*exons excluding UTRs

**distance to next gene

### Ways to enhance overall efficiency

In the described scheme, when new crosses were set up, one had to switch between fluorescence (to detect the transformation marker) and normal light (to determine sex) several times, which was a time-consuming procedure. To improve this situation considerably, we constructed and are testing new donors that use rescue of eye color by *vermilion*^+ ^as an indication of transformation [23,24]. The use of such a system will also facilitate stock-keeping.

Another way to enhance efficiency in future screens might be the establishment of donors that include an artificial maternal-effect selfish element, e.g. *Medea *[[25,26], see also Methods]. Such elements induce the death of all offspring of a female (maternal-lethal effect) except for those that have inherited the element (zygotic rescue). For example, a modified *piggyBac *donor element could incorporate a *Medea *element in tandem with the 3xP3-EGFP-marker. This modified donor element would be inserted at a chromosomal location tightly linked to an easily-scored recessive marker, such as the body-color mutation *black *[27]. In the P_1 _cross this donor strain (homozygous for wild-type body color) would be mated with a helper strain (homozygous mutant for *black*). The resulting P_2 _animals would carry one copy each of the helper, the donor and the mutant *black *allele. Moreover, the latter two would be located in *trans *at similar positions on homologous chromosomes. Such P_2 _hybrids would be mated with beetles that were *trans*-heterozygous for *black *and the *Medea*-containing donor element, or, if the P_2 _hybrid is female, they could instead be mated to homozygous *black *(non-helper, non-*Medea*) males. F_1 _offspring with black body-color then would arise only if they inherit the *black *chromosome from both parents. Because the *Medea*-tagged donor is arranged in *trans *with *black*, such *black *offspring do not carry a donor, and hence lack zygotic rescue activity of the *Medea *element. This leads to their death by the maternal-lethal effect of the element. Only if the donor has been remobilized to another genomic location can offspring carry both *black *alleles as well as the rescuing donor. Hence, black body-color in the offspring indicates a remobilization event. This design would be an elegant means to enhance the detection of new insertion lines by obviating the need for fluorescence detection. It would also simplify the stock-keeping of lethal insertions, since a *Medea *element tightly linked with the lethal insertion would constitute a type of balanced lethal.

## Conclusion

We have successfully established a method to conduct large-scale insertional mutagenesis screens in the beetle *Tribolium castaneum*. Using this method, we obtained several hundred lethal insertions as well as insertions producing enhancer-trap phenotypes. These lines have been made available to the research community.

## Methods

### Strains used

The *donor *strain used in this screen, Pig-19, carries a 3xP3-EGFP marked *piggyBac *element, pBac [3xP3-EGFPaf], that confers both, insertion-site-independent eye-specific EGFP expression, and donor-site-dependent muscle-specific EGFP expression [3]. We previously demonstrated that remobilization of the Pig-19 insertion results in G_1 _beetles lacking muscle-specific expression, but retaining eye-specific expression [3,11]. Thus, the loss of muscle-specific expression can be used to detect remobilization events. The *jumpstarter/helper *strain used in this screen, M26 [11], carries an X-chromosomal insertion of a 3xP3-DsRed marked *Minos *element (pMi [3xP3-DsRed; Dm'hsp70-pBac]) [9]. Both strains are in a white-eyed *pearl *mutant background to facilitate detection of eye-specific fluorescence.

### Generating new *piggyBac *insertions

We used a P_1_, P_2 _and F_1 _to F_4 _scheme to comply with the nomenclature of standard *Drosophila *F_1 _and F_3 _genetic screens, respectively (Figure 1). Donor remobilization occurred in the germline of the P_2 _generation, while new insertions and mutant homozygotes first appeared in the F_1 _and F_3 _generations, respectively. All crosses were carried out at 30-32°C. Virgin females were collected as pupae and stored at 23°C for up to six weeks prior to use. Insertional mutagenesis is described in detail in [11]. In summary, P_1 _mass-matings were set up between donor males and helper females (Figure 1A) and subcultured at intervals of two to three weeks. P_2 _offspring were collected as pupae and examined to verify the presence of both *piggyBac*-based donor (EGFP marker) and *Minos*-based helper (DsRed marker) constructs. Individual P_2 _virgin females were outcrossed to three *pearl *males each to ensure insemination (Figure 1B). The *piggyBac *donor element can be remobilized by *piggyBac *transposase activity in the germ line of the hybrid. New insertions were recognized in the F_1 _progeny by the loss of donor-site-dependent EGFP expression (i.e. muscle fluorescence) coupled with retention of insertion-site-independent EGFP expression (i.e. eye fluorescence). For each P_2 _outcross, a single F_1 _beetle carrying a new insertion was outcrossed once again to *pearl *to check for single insertions (based on 50% Mendelian segregation of the new insert) and to generate families for subsequent analysis. For stability of the new insertions, only individuals carrying a new insertion and lacking the helper element (i.e. DsRed negative) were chosen (Figure 1C). Additionally, depending on the new chromosomal location of *piggyBac*, a new insertion might show a novel enhancer-trap pattern. Even when a P_2 _cross produced multiple EGFP-positive offspring, only one F_1 _beetle was chosen for continued study in order to ensure independent origin of each new insertion. This was necessary because several offspring carrying the same insertion could appear within a P_2 _family as a result of a premeiotic remobilization event. For each F_1 _outcross, five female and three male F_2 _siblings were crossed to each other to establish new insertion strains and to enable testing for homozygous viability (F_2 _cross; Figure 1D). To accomplish the latter, we performed a number of single-pair F_3 _matings (Figure 1E) and analyzed their progeny for the presence of the donor element (see below).

### Statistical considerations

If an insertion mutant were homozygous viable, then (after positive marker selection) the progeny of the F_2 _cross would consist of a 1:2 ratio of homozygous to heterozygous beetles. Under the assumption of random sib-mating, 11.1% (1/3 × 1/3) of all F_3 _single-pair matings would be between two homozygous beetles, 44.4% [2× (1/3 × 2/3)] between one homozygous and one heterozygous beetle, and 44.4% (2/3 × 2/3) between two heterozygous beetles. This implies that about 55.5% (11.1% + 44.4%) of the single-pair matings (given a fully-viable insertion) would produce only EGFP-positive progeny (because at least one parent would be homozygous for the insertion). The remaining 44.4% would produce mixed progeny (i.e. approximately 75% EGFP positive and approximately 25% EGFP negative) because both parents would be heterozygous for the insertion. In contrast, for recessive lethal insertions, no homozygous beetles would be present in the F_3 _generation so all F_3 _crosses would produce mixed progeny. Thus, the presence of even a single EGFP-negative beetle in the F_4 _generation indicates heterozygosity of both parents, and the complete absence of EGFP-negative progeny indicates homozygosity of at least one parent. Depending on the distribution of the above-mentioned phenotypes, each single-pair mating was scored and assigned to one of five categories (see Figure 1F and Table 4 for details).

**Table 4:** Evaluation of F_3 _single-pair matings

Offspring of a single-pair mating	Interpretation/Result
No offspring, but parents alive at the time of evaluation	Indicates sterility of one or both parents
No offspring, but one or both parents dead at the time of evaluation	uninformative single-pair mating*
At least one EGFP-negative progeny	This indicates heterozygosity of both parents.
All progeny EGFP positive, at least 20 beetles present	This indicates homozygosity of one or both parents
All progeny EGFP positive, but less than 20 beetles present	uninformative single-pair mating*

*These single-pair matings were omitted from the overall evaluation (see Methods)

Since more than 40% of all single-pair matings were expected to produce mixed progeny (even if the insertion was fully viable) we analyzed a total of 20 single-pair matings before concluding that an insertion was lethal. On the other hand, since viable insertions were usually identified after evaluating just a few single-pair matings, we split the 20 crosses into two consecutive rounds to maximize throughput. The second round of single-pair matings would be set up only if an insertion were not clearly identified as *viable *after evaluating the first round (Table 5).

**Table 5:** Test for lethality and sterility

First round of F_3 _single-pair matings (SPM)		**Second round of F**_3 _**single-pair matings (SPM)**	
**Offspring**	**Phenotype**	**Offspring**	**Phenotype**
At least one SPM indicates homozygosity	Viable	At least one SPM (in total) indicates homozygosity	Viable
All informative* SPM indicate heterozygosity of both parents	Potentially lethal	All informative^* ^SPM indicate heterozygosity of both parents	Lethal
At least two SPM without any offspring but with living parents	Potentially sterile	Unable to find at least four SPM (in total) without any offspring but with living parents (method 1) OR unable to identify either a fertile homozygous female or a fertile homozygous male (method 2)	Sterile

After the first round of single-pair matings (SPM), all viable insertions were discarded (unless an enhancer trap was detected). All potentially lethal and potentially sterile lines were retested in a second round of single-pair matings.

*A single-pair mating is uninformative if it produces no offspring and one or both parents are dead, or less than 20 offspring are present and all of them are GFP-positive (see Table 4 and Methods)

The following potential errors could occur using this method to test for recessive lethality: (1) A homozygous-viable insertion mutant could be falsely judged homozygous lethal because all single-pair matings produced mixed progeny. This could occur if, by chance, all single-pair matings consisted of heterozygous beetles. The probability of such an occurrence is (^2^/_3_)^n ^(n = number of beetles tested), because two-thirds of all EGFP-positive F_3 _beetles carrying a viable insertion are heterozygous. For eight single-pair matings (number of test beetles = 16), this probability equals 0.15%. For 20 single-pair matings, the probability that all (40) test beetles selected from a homozygous-viable line are heterozygous, is only 9.0 × 10^-6^. Thus, evaluating 20 single-pair matings is sufficient to exclude false-positive lethal lines with a very high level of confidence. (2) A homozygous-lethal insertion (all F_2 _progeny are heterozygous) could be falsely identified as homozygous viable if, by chance, no EGFP-negative progeny are observed from a single-pair mating, even though 25% are expected. The probability of this happening when 20 progeny are analyzed is about 0.3% (0.75^n^; n = number of progeny screened). Because the probability of misdiagnosing a lethal insertion rises if fewer progeny are analyzed, single-pair matings yielding less than 20 progeny were not used to make inferences about the lethality of the insertion (= 'uninformative single-pair mating' in Table 4) unless some progeny were EGFP negative.

### Overcoming a negative X-chromosome bias

The fact that the helper insertion used in this work is X-linked imposed restrictions on the design of our crossing scheme. X-chromosomal insertions that were homozygous lethal or sterile could be obtained only if the following is considered: Because only new transformants that segregated away from the helper element were selected, hybrid females had to be used to set up P_2 _crosses in order to avoid bias against new X-linked insertions. Additionally, males with a new hemizygous X-linked lethal insertion would not survive and ones hemizygous for a new X-linked sterile insertion would be useless for generating a new stock. Hence, one could obtain X-linked lethal and sterile insertions only if female beetles carrying the donor element were used to set up the P_2 _as well as the F_1 _crosses. Therefore, we selected only female hybrids and used females carrying new insertions whenever possible.

### Efficiency of detecting new insertions

At least one new insertion was detected in about 30% of all P_2 _crosses when about 20 offspring were screened. The percentage of P_2 _crosses that yield new insertions can be greatly increased by screening a larger number of progeny per P_2 _cross. For a subset of P_2 _crosses we screened 100 progeny per cross, and found at least one new insertion in every case. In practice, about 10 - 30 P_2 _pupae were present when the P_2 _progeny were screened for new insertions. The decision to discard the larval P_2 _offspring when a new insertion could not be detected in the first attempt represented a compromise between the need to find at least one new insertion in each family and the aim to obtain a large number of independent insertions with limited resources in time and space.

### Determination of insertion sites

The genomic location of an insertion was determined by sequencing flanking DNA obtained by one of the following three polymerase chain reaction (PCR) -based methods: inverse PCR [28], universal PCR [3,29], or vectorette PCR [30]. The procedure for inverse PCR including primer design was adapted from 'Inverse PCR and Sequencing Protocol on 5 Fly Preps', Exelixis Pharmaceutical Corp (South San Francisco, California, USA) [22]. Following DNA isolation, approximately 1 μg of DNA was digested with *Sau*3A1, *BfU*C1, or *Ase*1 (for 5' iPCR) or *Hin*P1 (for 3' iPCR). Approximately 100 ng of digested DNA was then self-ligated to obtain circular DNA fragments, followed by two rounds of nested PCR. DNA templates (PCR products and/or cloned PCR products) were sequenced by Seqlab (Göttingen, Germany), Macrogen (Seoul, Korea), or using an ABI 3730 DNA sequencer (Sequencing and Genotyping Facility, Plant Pathology, Kansas State University, Manhattan, Kansas, USA). Data analysis was performed using Vector NTI^® ^software (Invitrogen, Carlsbad, California, USA). After trimming vector sequences, flanking DNA sequences were then searched (BLASTN) against *Tribolium castaneum *genome sequences at HGSC, Baylor College of Medicine http://www.hgsc.bcm.tmc.edu/projects/tribolium/, NCBI http://www.ncbi.nlm.nih.gov/genome/seq/BlastGen/BlastGen.cgi?taxid=7070 or BeetleBase http://beetlebase.org/. If the insertion was in a predicted gene (GLEAN set), a transcription unit (EST or cDNA) or region indicated by *Drosophila *BLAST or other gene prediction method as a potential gene, the predicted *Tribolium *gene was examined by BLAST analysis at FlyBase for the top *Drosophila *hit, and NCBI (nr database) to identify other potential orthologs. Insertion site sequences were deposited to NCBI (for accession numbers see Additional File 1) and also put - including the retrieved information - into GEKU-base (see below).

### *Medea *(maternal effect dominant embryonic arrest)

When hybrid females and *pearl *males (P_2 _generation) were crossed severe segregation distortion was observed: 98% of the progeny were EGFP positive, rather than the expected 50%. The DsRed marker however showed the expected 1:1 ratio (i.e. segregated independently of the EGFP marker). The unusual segregation of EGFP has been shown [11] to be the result of close *cis*-linkage (approximately 2 cM) of the maternally acting selfish gene *Medea *[25] with the Pig-19 donor insertion [3] on LG3. However, the segregation ratios of new insertions were affected only when the *piggyBac *element reinserted near the original donor insertion (representing a local hop).

### GEKU-base

All available information about the analyzed insertion lines can be found at a web-based database called GEKU-base http://www.geku-base.uni-goettingen.de. Information provided includes (if available) photographs and descriptions of enhancer traps and phenotypes, flanking sequences and chromosomal location, affected genes and their orthologs. GEKU-base also provides information on how to obtain insertion lines.

### EGFP and DsRed analysis

Marker-gene fluorescence was detected using a Nikon fluorescence stereomicroscope SMZ1500 (Nikon GmbH, Düsseldorf, Germany) at Göttingen and Erlangen, an Olympus SZX12 fluorescence stereomicroscope (Olympus Corporation, Tokyo, Japan), or a Leica MZ FLIII fluorescence stereomicroscope (Leica Microsystems Inc., Wetzlar, Germany). The filter sets used for EGFP expression were: [Göttingen: 470/40 nm excitation filter, 500 nm LP emission filter, and 495 nm beamsplitter], [Erlangen: 480/40 nm excitation filter, 510 nm emission filter, and 505 nm beamsplitter], [KSU: 480/40 nm excitation filter and 535/50 nm emission filter], [USDA: GFP Plus filter set (excitation filter: 480/40 nm, barrier filter: 510 nm)]. The filter sets used for DsRed expression were: [Göttingen: 546/12 nm excitation filter, 605/75 nm emission filter, and 560 nm beamsplitter], [Erlangen: 565/30 excitation filter, 620/60 nm emission filter, and 585 nm beamsplitter], [KSU: 545/30 excitation filter and 620/60 emission filter], [USDA: TXR TEXAS RED filter set (excitation filter: 560/40 nm, barrier filter: 610 nm)]. To detect enhancer-trap patterns in embryos, we dechorionated embryos derived from F_3_-crosses.

### Location of insertion lines shown in Figure 2

Gene names refer to respective *Drosophila *orthologs. The line E00321 is homozygous lethal and carries an insertion in *lethal (2) giant larvae *(Figure 2A). The line E00713 is homozygous viable and carries an insertion 149-bp upstream of the 5' end of GLEAN_03347, *Glutatione S transferase*, (Figure 2B). The homozygous viable line G01004 carries an insertion near *Ultrabithorax *(Figure 2C). The homozygous viable line G04717 carries an insertion near *lame duck *(Figure 2D). The line KT1539 is homozygous lethal and the insertion site is near the gene *pointed *(Figure 2E). The homozygous lethal line KS030 bears an insertion in an intron of *lozenge *(Figure 2F). The KS406 line is homozygous viable and carries an insertion in an intron of GLEAN_00277 which shows similarity to genes encoding protein tyrosine phosphatases. Other genes in the vicinity of this insertion are *Fgf8 *or *Or48 *(Figure 2G). The homozygous viable line MH30a has an insertion near *female sterile (2) Ketel *(Figure 2H). The line E00916 is homozygous lethal and carries an insertion in an exon of GLEAN_08270 (*Drosophila *ortholog: *Cyclin D*) (Figure 2I). The G08519 insertion is located in the first intron of *proboscipedia *(Figure 2J). The KT1096 insertion is in an intron of the *pecanex *ortholog (Figure 2K). The E03501 insertion is in an intron of the *Tribolium *ortholog of *Ftz-F1 *(Figure 2L).

## Abbreviations

bp: base pair; CDS: coding sequence; cM: centiMorgan; EGFP: enhanced green fluorescent protein; GEKU: Göttingen, Erlangen, KSU, USDA; KSU: Kansas State University; L1: first larval stage; LG: linkeage group; Mb: Megabase; MEDEA: maternal arrest dominant embryonic arrest; RNAi: RNA interference; SPM: single-pair mating; USDA: United States Department of Agriculture; UTR: untranslated region

## Authors' contributions

JT, JS, MDL and TDS were involved in setting up and evaluating the beetle crosses, screening for enhancer traps and determining the genomic location of insertion sites. JT drafted the manuscript. EAW, RWB, MK, GB, MDL, TDS and SJB conceived of the study, participated in its design and coordination, and helped to draft the manuscript. All authors read and approved the final manuscript.

## Supplementary Material

Additional file 1 Gene bank accession numbers of integration site sequences. Click here for file
