# Supplementary material for: Large-scale insertional mutagenesis of a coleopteran stored grain pest, the red flour beetle Tribolium castaneum, identifies embryonic lethal mutations and enhancer traps
Source: BMC Biol. 2009 Nov 5;7:73. doi: 10.1186/1741-7007-7-73 (PMC2779179; doi:10.1186/1741-7007-7-73)
Supplement: Additional file 1 — Gene bank accession numbers of integration site sequences. [file 1741-7007-7-73-S1.pdf]

Additional File 1.
Gene bank accession numbers of integration site sequences.

dbGSS_Id	User_Id	GenBank_Accn
26237633	E00108left	GS375779
26237634	E00212left	GS375780
26237635	E00213left	GS375781
26237636	E00218left	GS375782
26237637	E00221left	GS375783
26237638	E00310left	GS375784
26237639	E00315left	GS375785
26237640	E00321left	GS375786
26237641	E00323left	GS375787
26237642	E00324left	GS375788
26237643	E00414left	GS375789
26237644	E00416left	GS375790
26237645	E00419left	GS375791
26237646	E00505left	GS375792
26237647	E00521left	GS375793
26237648	E00622left	GS375794
26237649	E00703left	GS375795
26237650	E00713left	GS375796
26237651	E00819left	GS375797
26237652	E00822left	GS375798
26237653	E00904left	GS375799
26237654	E00905left	GS375800
26237655	E00916left	GS375801
26237656	E00923left	GS375802
26237657	E01004left	GS375803
26237658	E01014left	GS375804
26237659	E01101left	GS375805
26237660	E01106left	GS375806
26237661	E01212left	GS375807
26237662	E01213left	GS375808
26237663	E01508left	GS375809
26237664	E01511left	GS375810
26237665	E01512left	GS375811
26237666	E01702left	GS375812
26237667	E01709left	GS375813
26237668	E02007left	GS375814
26237669	E02013left	GS375815
26237670	E02101left	GS375816
26237671	E02201left	GS375817
26237672	E02309left	GS375818
26237673	E02401left	GS375819
26237674	E02408left	GS375820
26237675	E02501left	GS375821
26237676	E02601left	GS375822
26237677	E02805left	GS375823
26237678	E02907left	GS375824
26237679	E03004left	GS375825
26237680	E03005left	GS375826
26237681	E03207left	GS375827

26237682	E03411left	GS375828
26237683	E03501left	GS375829
26237684	E03503left	GS375830
26237685	E03504left	GS375831
26237686	E03801left	GS375832
26237687	E04201left	GS375833
26237688	E04408left	GS375834
26237689	E04411left	GS375835
26237690	E04601left	GS375836
26237691	E04701left	GS375837
26237692	E05016left	GS375838
26237693	E05017left	GS375839
26237694	E05107left	GS375840
26237695	E05201left	GS375841
26237696	E00311right	GS375842
26237697	E00822right	GS375843
26237698	E03608right	GS375844
26237699	E04410right	GS375845
26237700	E04809right	GS375846

26237773	A028right	GS375919
26237774	HC198right	GS375920
26237775	HC202right	GS375921
26237776	HC208right	GS375922
26237777	KL043right	GS375923
26237778	KL063right	GS375924
26237779	KL224right	GS375925
26237780	KS030right	GS375926
26237781	KS137right	GS375927
26237782	KS209right	GS375928
26237783	KS234right	GS375929
26237784	KS249right	GS375930
26237785	KS283right	GS375931
26237786	KS294right	GS375932
26237787	KS374right	GS375933
26237788	KT045right	GS375934
26237789	KT054right	GS375935
26237790	KT087right	GS375936
26237791	KT1021right	GS375937
26237792	KT1023right	GS375938
26237793	KT1041right	GS375939
26237794	KT1096right	GS375940
26237795	KT1100right	GS375941
26237796	KT1142right	GS375942
26237797	KT1161right	GS375943
26237798	KT1183right	GS375944
26237799	KT1194right	GS375945
26237800	KT1216right	GS375946
26237801	KT1236right	GS375947
26237802	KT1239right	GS375948
26237803	KT1261right	GS375949
26237804	KT1264right	GS375950
26237805	KT1269right	GS375951
26237806	KT1281right	GS375952
26237807	KT1299right	GS375953
26237808	KT1356right	GS375954
26237809	KT139right	GS375955

26237810	KT144right	GS375956
26237811	KT1458right	GS375957
26237812	KT149right	GS375958
26237813	KT1530right	GS375959
26237814	KT1537right	GS375960
26237815	KT1539right	GS375961
26237816	KT1571right	GS375962
26237817	KT158right	GS375963
26237818	KT1597right	GS375964
26237819	KT160right	GS375965
26237820	KT161right	GS375966
26237821	KT203right	GS375967
26237822	KT214right	GS375968
26237823	KT221right	GS375969
26237824	KT224right	GS375970
26237825	KT292right	GS375971
26237826	KT323right	GS375972
26237827	KT351right	GS375973
26237828	KT359right	GS375974
26237829	KT375right	GS375975
26237830	KT397right	GS375976
26237831	KT430right	GS375977
26237832	KT443right	GS375978
26237833	KT450right	GS375979
26237834	KT469right	GS375980
26237835	KT487right	GS375981
26237836	KT501right	GS375982
26237837	KT508right	GS375983
26237838	KT529right	GS375984
26237839	KT548right	GS375985
26237840	KT551right	GS375986
26237841	KT561right	GS375987
26237842	KT566right	GS375988
26237843	KT569right	GS375989
26237844	KT616right	GS375990
26237845	KT619right	GS375991
26237846	KT653right	GS375992
26237847	KT693right	GS375993
26237848	KT709right	GS375994
26237849	KT748right	GS375995
26237850	KT775right	GS375996
26237851	KT850right	GS375997
26237852	KT891right	GS375998
26237853	KT911right	GS375999
26237854	KT915right	GS376000
26237855	KT955right	GS376001
26237856	KT966right	GS376002
26237857	KT979right	GS376003
26237858	KTR1702right	GS376004
26237859	KTR1715right	GS376005
26237860	KTR1735right	GS376006
26237861	KTR1804right	GS376007
26237862	KTR1816right	GS376008
26237863	KTR1833right	GS376009
26237864	MH22bright	GS376010
26237865	MH30aright	GS376011
26237866	MH30peaRright	GS376012

26237867	MH40aright	GS376013
26237868	MH46aRright	GS376014
26237869	HC004left	GS376015
26237870	HC157left	GS376016
26237871	KS217left	GS376017
26237872	KS231left	GS376018
26237873	KS255left	GS376019
26237874	KS256left	GS376020
26237875	KS265left	GS376021
26237876	KS342left	GS376022
26237877	KS355left	GS376023
26237878	KS406left	GS376024
26237879	KT017left	GS376025
26237880	KT033left	GS376026
26237881	KT045left	GS376027
26237882	KT058left	GS376028
26237883	KT065left	GS376029
26237884	KT076left	GS376030
26237885	KT1087-Xleft	GS376031
26237886	KT1100left	GS376032
26237887	KT1239left	GS376033
26237888	KT127left	GS376034
26237889	KT1281left	GS376035
26237890	KT148left	GS376036
26237891	KT217left	GS376037
26237892	KT241left	GS376038
26237893	KT372left	GS376039
26237894	KT418left	GS376040
26237895	KT580left	GS376041
26237896	KT713left	GS376042
26237897	KT777left	GS376043

26239002	G00211left	GS377147
26239003	G00216left	GS377148
26239004	G00220left	GS377149
26239005	G00304left	GS377150
26239006	G00310left	GS377151
26239007	G00312left	GS377152
26239008	G00410left	GS377153
26239009	G00514left	GS377154
26239010	G00611left	GS377155
26239011	G00617left	GS377156
26239012	G00805left	GS377157
26239013	G00912left	GS377158
26239014	G00915left	GS377159
26239015	G00919left	GS377160
26239016	G01004left	GS377161
26239017	G01005left	GS377162
26239018	G01022left	GS377163
26239019	G01123left	GS377164
26239020	G01205left	GS377165
26239021	G01207left	GS377166
26239022	G01217left	GS377167
26239023	G01308left	GS377168
26239024	G01316left	GS377169
26239025	G01318left	GS377170
26239026	G01320left	GS377171

26239027	G01412left	GS377172
26239028	G01505left	GS377173
26239029	G01516left	GS377174
26239030	G01517left	GS377175
26239031	G01702left	GS377176
26239032	G01724left	GS377177
26239033	G01809left	GS377178
26239034	G01821left	GS377179
26239035	G01901left	GS377180
26239036	G01910left	GS377181
26239037	G01915left	GS377182
26239038	G01922left	GS377183
26239039	G02004left	GS377184
26239040	G02009left	GS377185
26239041	G02010left	GS377186
26239042	G02014left	GS377187
26239043	G02111left	GS377188
26239044	G02205left	GS377189
26239045	G02210left	GS377190
26239046	G02218left	GS377191
26239047	G02220left	GS377192
26239048	G02306left	GS377193
26239049	G02308left	GS377194
26239050	G02404left	GS377195
26239051	G02408left	GS377196
26239052	G02418left	GS377197
26239053	G02504left	GS377198
26239054	G02505left	GS377199
26239055	G02508left	GS377200
26239056	G02607left	GS377201
26239057	G02613left	GS377202
26239058	G02614left	GS377203
26239059	G02617left	GS377204
26239060	G02706left	GS377205
26239061	G02716left	GS377206
26239062	G02805left	GS377207
26239063	G02919left	GS377208
26239064	G03001left	GS377209
26239065	G03121left	GS377210
26239066	G03204left	GS377211
26239067	G03311left	GS377212
26239068	G03318left	GS377213
26239069	G03323left	GS377214
26239070	G03418left	GS377215
26239071	G03508left	GS377216
26239072	G03720left	GS377217
26239073	G03802left	GS377218
26239074	G03822left	GS377219
26239075	G03901left	GS377220
26239076	G03914left	GS377221
26239077	G03920left	GS377222
26239078	G04105left	GS377223
26239079	G04119left	GS377224
26239080	G04206left	GS377225
26239081	G04321left	GS377226
26239082	G04504left	GS377227
26239083	G04508left	GS377228

26239084	G04520left	GS377229
26239085	G04521left	GS377230
26239086	G04614left	GS377231
26239087	G04619left	GS377232
26239088	G04622left	GS377233
26239089	G04623left	GS377234
26239090	G04717left	GS377235
26239091	G04909left	GS377236
26239092	G05201left	GS377237
26239093	G05211left	GS377238
26239094	G05215left	GS377239
26239095	G05324left	GS377240
26239096	G05402left	GS377241
26239097	G05416left	GS377242
26239098	G05418left	GS377243
26239099	G05514left	GS377244
26239100	G05602left	GS377245
26239101	G05614left	GS377246
26239102	G05623left	GS377247
26239103	G05809left	GS377248
26239104	G06002left	GS377249
26239105	G06201left	GS377250
26239106	G06213left	GS377251
26239107	G06614left	GS377252
26239108	G06714left	GS377253
26239109	G06806left	GS377254
26239110	G06902left	GS377255
26239111	G07014left	GS377256
26239112	G07021left	GS377257
26239113	G07109left	GS377258
26239114	G07122left	GS377259
26239115	G07219left	GS377260
26239116	G07309left	GS377261
26239117	G07313left	GS377262
26239118	G07405left	GS377263
26239119	G07411left	GS377264
26239120	G07418left	GS377265
26239121	G07421left	GS377266
26239122	G07506left	GS377267
26239123	G07510left	GS377268
26239124	G07601left	GS377269
26239125	G07615left	GS377270
26239126	G07723left	GS377271
26239127	G07806left	GS377272
26239128	G07809left	GS377273
26239129	G07821left	GS377274
26239130	G07911left	GS377275
26239131	G08102left	GS377276
26239132	G08112left	GS377277
26239133	G08117left	GS377278
26239134	G08204left	GS377279
26239135	G08216left	GS377280
26239136	G08404left	GS377281
26239137	G08408left	GS377282
26239138	G08412left	GS377283
26239139	G08415left	GS377284
26239140	G08507left	GS377285

26239141	G08521left	GS377286
26239142	G08602left	GS377287
26239143	G08611left	GS377288
26239144	G08710left	GS377289
26239145	G09012left	GS377290
26239146	G09013left	GS377291
26239147	G09101left	GS377292
26239148	G09104left	GS377293
26239149	G09206left	GS377294
26239150	G09301left	GS377295
26239151	G09313left	GS377296
26239152	G09407left	GS377297
26239153	G09807left	GS377298
26239154	G09808left	GS377299
26239155	G09821left	GS377300
26239156	G09914left	GS377301
26239157	G09917left	GS377302
26239158	G09922left	GS377303
26239159	G10003left	GS377304
26239160	G10011left	GS377305
26239161	G10013left	GS377306
26239162	G10024left	GS377307
26239163	G10119left	GS377308
26239164	G10205left	GS377309
26239165	G10211left	GS377310
26239166	G10215left	GS377311
26239167	G10216left	GS377312
26239168	G10302left	GS377313
26239169	G10321left	GS377314
26239170	G10401left	GS377315
26239171	G10411left	GS377316
26239172	G10424left	GS377317
26239173	G10507left	GS377318
26239174	G10518left	GS377319
26239175	G10519left	GS377320
26239176	G10621left	GS377321
26239177	G10803left	GS377322
26239178	G10811left	GS377323
26239179	G10905left	GS377324
26239180	G10909left	GS377325
26239181	G11014left	GS377326
26239182	G11021left	GS377327
26239183	G11022left	GS377328
26239184	G11111left	GS377329
26239185	G11116left	GS377330
26239186	G11122left	GS377331
26239187	G11319left	GS377332
26239188	G11323left	GS377333
26239189	G11410left	GS377334
26239190	G11514left	GS377335
26239191	G11515left	GS377336
26239192	G11605left	GS377337
26239193	G11701left	GS377338
26239194	G11710left	GS377339
26239195	G11721left	GS377340
26239196	G11723left	GS377341
26239197	G11904left	GS377342

26239198	G11905left	GS377343
26239199	G12009left	GS377344
26239200	G12106left	GS377345
26239201	G12109left	GS377346
26239202	G12211left	GS377347
26239203	G12311left	GS377348
26239204	G12312left	GS377349
26239205	G12322left	GS377350
26239206	G12402left	GS377351
26239207	G12403left	GS377352
26239208	G12416left	GS377353
26239209	G12420left	GS377354
26239210	G12424left	GS377355
26239211	G12524left	GS377356
26239212	G12601left	GS377357
26239213	G12606left	GS377358
26239214	G12616left	GS377359
26239215	G12702left	GS377360
26239216	G12918left	GS377361
26239217	G13015left	GS377362
26239218	G13024left	GS377363
26239219	G13107left	GS377364
26239220	G13201left	GS377365
26239221	G13222left	GS377366
26239222	G13316left	GS377367
26239223	G13402left	GS377368
26239224	G13407left	GS377369
26239225	G13701left	GS377370
26239226	G09621left	GS377371
26868763	G05020left	GS504207
26868764	G06602left	GS504208
26868765	G08519left	GS504209
26868766	G10403left	GS504210
26868767	G10405left	GS504211
26868768	G13104left	GS504212
